# Influence of Drying Methods on Volatile Aroma Compounds and Sensory Properties of Olive Leaf Herbal Tea

**DOI:** 10.3390/foods15030496

**Published:** 2026-02-01

**Authors:** Gianluca Tripodi, Marco Torre, Antonella Verzera, Fabrizio Cincotta

**Affiliations:** 1 Department of Human Sciences and Promoting of the Quality of Life, San Raffaele Telematic University Rome, Via Val Cannuta 247, 00166 Rome, Italy; gianluca.tripodi@uniroma5.it; 2 Department of Veterinary Sciences, University of Messina, Viale G. Palatucci, 98168 Messina, Italy or marco.torre@unito.it (M.T.); fabrizio.cincotta@unime.it (F.C.); 3 Department of Agricultural, Forestry and Food Sciences—DISAFA, University of Turin, Via Verdi 8, 10124 Torino, Italy

**Keywords:** *Olea europaea* L. leaves, leaf herbal tea, drying processes, air drying, microwave drying, volatile compounds, sensory analysis, consumer acceptability

## Abstract

Olive leaves, a by-product of the olive oil industry, represent an interesting underutilized raw material for the preparation of herbal teas. However, processing conditions, particularly drying methods, may strongly influence their chemical and sensory quality. This study aimed to evaluate the effect of air drying (AD) and microwave drying (MWD) on the phenolic content, antioxidant capacity, volatile aroma compounds, sensory profile, and consumers’ acceptability of olive leaf herbal teas. Olive leaves were subjected to AD (50 °C, 3 h) and MWD (400 W, 4 min), and infusions were prepared. Total phenolic content (TPC) and antioxidant capacity (AC) were evaluated spectrophotometrically, volatile compounds were analyzed by HS-SPME-GC-MS, and sensory characteristics were assessed through descriptive sensory analysis and consumers’ acceptability test. MWD significantly increased TPC compared to AD; however, this increase was not proportionally reflected in AC. The drying methods influenced the volatile profile of herbal teas, with AD showing a higher amount of alcohols, esters, and terpenes associated with green and floral notes, whereas MWD showed a major content of aldehydes and ketones linked to fruity notes. Sensory analysis confirmed these differences; moreover, MWD herbal teas were more bitter and astringent, and consumer tests showed a clear preference for herbal teas produced from AD leaves. Overall, the results highlight the key role of drying methods in shaping the chemical and sensory attributes of olive leaf herbal tea, suggesting air drying to be the most suitable process for producing a sensorially acceptable product.

## 1. Introduction

The olive tree (*Olea europaea* L.) is among the most cultivated fruit trees worldwide [1]. In recent decades, the increasing global demand for olive oil and table olives has led to extensive olive groves and a constantly growing stream of by-products, including leaves from pruning and harvesting [2]. Olive leaves, long regarded as mere agricultural waste, represent a substantial biomass currently underutilized.

Olive leaves are rich in bioactive compounds. They contain a high concentration of phenolic compounds, primarily secoiridoids such as the major compound oleuropein, as well as flavonoids, phenylethanoids, hydroxycinnamic acids, iridoids, and triterpenic acids [3,4,5,6,7]. In vitro and in vivo studies have highlighted antioxidant, anti-inflammatory, antihypertensive, and metabolic effects attributable to olive leaf phenolics and related molecules [8,9,10,11,12]. For example, supplementation with olive-leaf-derived phenolic extracts has been associated with improvements in cardiovascular risk markers, including blood pressure and lipid profile [13,14]. Given the high concentration of bioactive molecules in olive leaves, often higher than in fruits or olive oil, and the ability to compete, in terms of antioxidant capacity, with synthetic antioxidants (e.g., BHT, BHA), they represent a natural and sustainable resource for functional applications in food and beyond [5,15].

Several studies have investigated the use of olive leaf for the preparation of herbal tea, mainly focusing on their phenolic composition [16,17,18], anti-diabetic properties [19,20], effects on lipid metabolism [21] and haematological health [22].

Considering the growing emphasis on sustainability and circular economy, valorising agricultural by-products is increasingly recognized as a priority. The use of olive leaves, a highly available and low-cost residue of the olive oil industry, offers a strategic opportunity to valorise waste into value-added products. Among potential applications, the development of herbal teas is particularly attractive. Compared to extracts, infusions allow a gentle, consumer-friendly way to intake bioactive compounds, aligning with current consumer trends toward natural, plant-based, functional beverages [23]. Indeed, the herbal tea market in Europe has experienced steady growth, fuelled by increased consumer demand for natural, health-promoting beverages [24]. This creates a favourable context for novel ingredients such as olive leaf-based infusions, especially when supported by functional performance and sustainability benefits.

Processing methods, particularly drying techniques, play a critical role in determining the phytochemical profile, aroma, sensory profile and overall quality of herbal teas [25,26,27]. Different drying procedures can significantly affect the amount of phenolic compounds and volatile aroma precursors in olive leaves [6,28,29,30]. For olive leaves, studies have shown that non-conventional drying methods such as microwave drying (MWD) may outperform traditional air drying (AD) in preserving or even enhancing the content of oleuropein and other phenolics. For instance, MWD under optimized conditions resulted in higher phenolic retention compared to several other drying techniques [6,31]. Conversely, freeze-drying, while effective in stabilizing certain compounds, may show larger losses in oleuropein upon subsequent storage or defrosting, due to enzymatic degradation [32,33,34]. This suggests that the selection and optimisation of the drying process represent a key step in obtaining a high-quality herbal tea with preserved aroma and bioactive properties.

Some studies have evaluated the effects of different drying processes on olive leaves intended for the production of herbal teas. For example, treatments such as boiling in a steam cooker, hot air, room temperature and infrared radiation have been considered [18,20,35]. However, these studies report on chemical composition, especially polyphenols, and colour, but do not evaluate the effects of drying processes on volatile aroma compounds, sensory characteristics and consumer acceptability of olive leaf herbal teas.

Given this background, this study aims to evaluate the influence of conventional air drying (AD) and microwave drying (MWD), on the phenolic content, volatile aroma compounds, sensory profile, and consumers’ acceptability of olive leaf herbal teas. Our goal was to establish whether olive leaves, processed under optimized conditions, can deliver a final product that is both functionally rich and sensorially appealing, thereby contributing to the valorisation of this olive industry by-product and to the diversification of the herbal tea market.

## 2. Materials and Methods

### 2.1. Sample Collection and Drying Processes

Fresh leaves of *Olea europaea* L. cv. Nocellara messinese were harvested randomly from an olive grove located in the province of Messina, Sicily, Italy, in October 2025. Following this, the leaves were immediately transported to the laboratory where they were cleaned with cold water to remove surface impurities, split into two batches, and subjected to Air Drying (AD) and Microwave Drying (MWD), respectively.

The drying procedures were based on the methods previously described by Cincotta et al. (2025) [26], with adjustments tailored to the present study. For air drying (AD), sets of 50 leaves were arranged on the trays of a forced-air dryer (Armfield Ltd., UOP8 model, Hampshire, UK) and maintained at 50 °C for 3 h, using an air flow of 1.5 m/s. For microwave drying (MWD), 30 g of fresh leaves were well spaced, to avoid overlapping, on a ceramic support inside a microwave unit (LG Electronics Inc., MH8265DPS.CB1QUESD model, Amstelveen, The Netherlands) and exposed to 400 W for 4 min. A temperature of ~51 °C was recorded at the end of MWD process. The selected operating parameters, temperature, duration, and microwave power, followed earlier optimization trials reported by Cincotta et al. (2025) [26], and were chosen to ensure optimal sensory properties, as confirmed in preliminary evaluations by a trained sensory panel. The initial moisture content of the leaves (% wet basis) was approximately 49 ± 0.2%, and drying was carried out until a moisture reduction of 98% was achieved (final moisture 1.9 ± 0.03%). Moisture was tracked by periodically weighing the samples with an analytical balance (Shimadzu, ATX224R, Kyoto, Japan, precision 0. 1 mg). All drying processes were conducted in triplicate.

### 2.2. Herbal Tea Preparation

The preparation of olive leaf herbal tea followed the same protocol used by Cincotta et al. (2025) [26]. 3 g of finely chopped dried leaves were infused for 10 min in 120 mL of mineral water at 95 °C. The solution was then filtered using standard filter paper (Munktell & Filtrak, Barenstein, Germany) and, for chemical analysis, cooled to room temperature. All infusions were prepared and analyzed in triplicate to ensure repeatability. The obtained herbal tea was then used for the following analysis.

### 2.3. Total Phenolic Content and Antioxidant Capacity

The total polyphenol content (TPC) and antioxidant capacity (AC) of olive leaf herbal tea were determined in accordance with the protocol described by Vinci et al. (2022) [36]. Spectrophotometric determinations were performed using the Folin–Ciocalteu method and the DPPH assay, respectively.

### 2.4. Volatile Aroma Compound Analysis

The volatile aroma compounds of olive leaf herbal tea were analyzed by headspace solid-phase microextraction coupled with gas chromatography tandem mass spectrometry (HS-SPME-GC-MS) following the procedure reported by Cincotta et al. (2025) [26]. For each analysis, 18 mL of infusion was transferred into a 40 mL glass vial, added with 4 g of NaCl, and allowed to equilibrate at 30 °C for 30 min. A DVB/CARB/PDMS SPME fiber was then exposed to the headspace for 30 min, followed by thermal desorption in the GC injector at 260 °C for 3 min. GC-MS analysis was performed by a Shimadzu GC-2010 Plus system coupled with a TQMS 8040 triple-quadrupole mass spectrometer (Shimadzu, Milan, Italy), equipped with a Vf-Wax-ms capillary column (60 m × 0.25 mm i.d., 0.25 μm film thickness). Chromatographic separation was achieved under the following conditions: injector temperature 260 °C; splitless mode; oven program starting at 45 °C (held for 5 min), increasing to 110 °C at 5 °C/min, then to 260 °C at 20 °C/min; helium as the carrier gas at a constant flow of 1 mL/min; transfer line temperature set to 250 °C. Mass spectra were collected over an *m*/*z* interval of 40–400 at a scan rate of 1250 amu/s. Volatile compound identification relied on comparison with the NIST’24 mass spectral library (NIST/EPA/NIH, Wiley, Gaithersburg, MD, USA) and the FFNSC 3.0 database, confirmation through analytical standards when available, calculation of linear retention indices (LRIs) using the Van den Dool and Kratz approach, and consultation of published reference data. Results were reported as relative peak area percentages, considering a peak area of 0.01% as the exclusion limit value.

### 2.5. Qualitative Descriptive Analysis

The qualitative descriptive analysis (QDA) of the olive leaf herbal teas was conducted following the approach adapted from Cincotta et al. (2025) [26]. All participants provided informed consent in accordance with the ethical principles of the Declaration of Helsinki. A sensory panel of eight judges completed a four-week training period in accordance with ISO 8586:2023 guidelines [37]. During the initial training sessions, a broad set of sensory attributes was proposed, from which fifteen were ultimately chosen based on their frequency of citation (>60%).

Clear definitions were established for each descriptor to ensure shared understanding among panelists. The descriptors were then validated using specific reference standards, allowing panel members to calibrate their evaluations and maintain consistency throughout the experimental sessions. Sensory intensities were scored on a nine-point scale, ranging from 1 (absence of sensation) to 9 (extremely intense).

Sessions were conducted between 10:00 and 12:00 a.m. in individual sensory booths under white lighting. Samples order was randomized for each judge and session to minimize positional and carryover effects ensuring three independent evaluations per judge for each sample. Water and unsalted crackers were provided to cleanse the palate between samples. The samples were analyzed at a temperature of 80–85 °C, without added substances such as sugar and lemon. All evaluations were recorded using the data acquisition software (FIZZ Byosistemes, version 2.00 M, Couternon, France).

### 2.6. Consumers’ Acceptability Test

The consumers’ acceptability assessment was carried out with a group of 78 individuals (35 males and 43 females) between 24 and 60 years of age. Participants were recruited among students and staff of the Department of Veterinary Sciences at the University of Messina through the convenience sampling method. Participants were asked about their normal tea consumption, such as frequency, use of sweeteners or lemon, temperature preference, and personal sensitivity to bitter products. Involvement in the study was entirely voluntary.

The samples were served randomly at a temperature of 80–85 °C, without the addition of other substances (sugar and lemon), between samples, water at room temperature and unsalted crackers were served to clean the mouth.

Participants evaluated the samples in terms of color, aroma, taste, and overall liking using a nine-point hedonic scale, where 1 indicated extreme dislike, and 9 indicated extreme appreciation [26].

### 2.7. Statistical Analysis

XLStat software, version 2024.1 (Addinsoft, New York, NY, USA) for the statistical elaboration of the data. A one-way analysis of variance (ANOVA) followed by Duncan’s multiple range test was applied to both chemical and sensory data at a 95% confidence level. Principal component analysis (PCA) was used to analyze volatile aroma compounds data [38].

## 3. Results and Discussions

### 3.1. Total Phenolic Content and Antioxidant Activity

**Figure 1:**
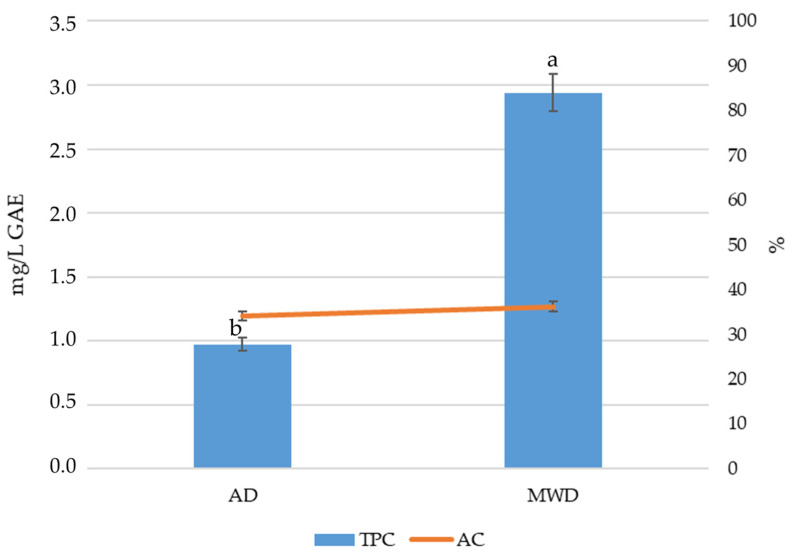
Total phenolic content (TPC) and antioxidant capacity (AC) of olive leaf herbal teas. AD: herbal tea from air dried olive leaf. MWD: herbal tea from microwave dried olive leaf. Different letters indicate statistically significant differences at *p* < 0.05 by Duncan’s test.

Figure 1 reports the total phenolic content (TPC) and antioxidant capacity (AC) of olive leaf herbal teas prepared from air dried (AD) and microwave dried (MWD) leaves.

MWD herbal teas showed a higher TPC (~3.0 mg/L GAE) than AD ones (~1.0 mg/L GAE). In contrast, the AC, expressed as the percentage inhibition of the DPPH radical, increased to a limited extent, from ~33% in AD to about 37% in MWD. These results indicate that, although MWD significantly enhanced the extraction of phenolic compounds, the increase in total phenolic content (TPC) did not result in a proportional enhancement of antioxidant capacity (AC).

The significant increase in TPC observed in MWD herbal teas is likely attributable to the rapid volumetric heating induced by microwaves, which disrupts cellular and subcellular structures, facilitating the release of bound phenolic compounds, glycosides, or conjugates that are less accessible under milder drying conditions. This interpretation is consistent with previous studies reporting enhanced TPC in microwave-assisted extraction (MAE) of olive leaves compared to conventional methods, owing to the more efficient disruption of cell walls and subsequent liberation of phenolics such as oleuropein [31,39,40].

Despite the clear TPC increase, the slight increase in AC indicates that not all extracted phenolics contribute equally to the antioxidant response in the DPPH assay. This trend could be related to the fact that microwave drying could selectively release compounds with low scavenging or redox potential, including polymerized, oxidized, or structurally altered phenolics [41,42]. Moreover, differences in assay sensitivity must be considered; the Folin–Ciocalteu method used for TPC quantification detects reducing substances broadly, whereas the AC assay selectively reflects compounds capable of neutralizing free radicals. Consequently, a higher TPC does not necessarily reflect a proportionally higher measured antioxidant capacity [43]. This phenomenon aligns with previous reports indicating that olive-leaf drying conditions can significantly affect both phenolic quantity and quality, including the preservation or degradation of major bioactive compounds such as oleuropein [35].

### 3.2. Volatile Aroma Compounds

Table 1 reports the volatile aroma compounds of olive leaf herbal teas produced with AD and MWD leaves.

**Figure 2:**
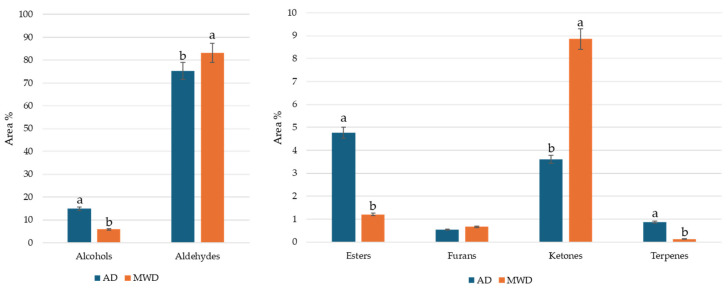
Volatile percentage composition for class of substances of olive leaf herbal teas. AD: herbal tea from air dried olive leaf. MWD: herbal tea from microwave dried olive leaf. Different letters in the same class of substances indicate statistically significant differences at *p* < 0.05 by Duncan’s test.

Aldehydes were the chemical class of substances most represented, followed by alcohols, ketones, esters, furans, and terpenes. Statistically significant differences were observed between the two treatments for most of the chemical classes. MWD treatment determined an increase in aldehydes and ketones content, whereas a higher content of alcohol and esters was observed in AD samples (Figure 2).

Across both treatments, aldehydes accounted for 75.29% in AD samples and 83.21% in MWD samples. Nonanal was the most abundant compound in both treatments (44.44% AD; 43.73% MWD), followed by octanal (6.74% AD; 14.03% MWD) and hexanal (2.83% AD; 5.54% MWD). These aldehydes are associated with citrus and orange peel notes, which are key contributors to the characteristic aroma of olive-derived products. Similar findings have been reported by Zhang et al. (2020) [44], who identified nonanal, hexanal, and benzaldehyde as major aroma-active compounds in Chinese olive leaf herbal tea.

The alcohols constituted the second most abundant class of volatile compounds in AD samples (14.92%), dominated by (*Z*)-3-hexen-1-ol (5.10%) and 3-methyl-1-butanol (2.75%), imparting fresh and green notes. In contrast, MWD samples exhibited a marked reduction in alcohols (5.86%) and esters (1.21%), while ketones increased substantially (8.86% vs. 3.61% in AD). Among ketones, 6-methyl-5-hepten-2-one (5.35% MWD) contributed citrus and fruity notes. Terpenes were detected at low levels, with eucalyptol and β-cyclocitral more abundant in AD samples, suggesting thermal degradation during MWD.

The differences in the volatile profiles between AD and MWD highlight the technological role of drying in shaping sensory attributes. AD preserved compounds associated with fresh, green, and fruity notes (C6 alcohols and esters), whereas MWD promoted aldehyde and ketone formation, resulting in a more citrus-oriented aroma. These trends align with previous reports indicating that microwave-assisted drying accelerates oxidative and thermal reactions, enhancing carbonyl compounds while reducing C6 alcohols and terpenes [25,26,27,45,46].

**Figure 3:**
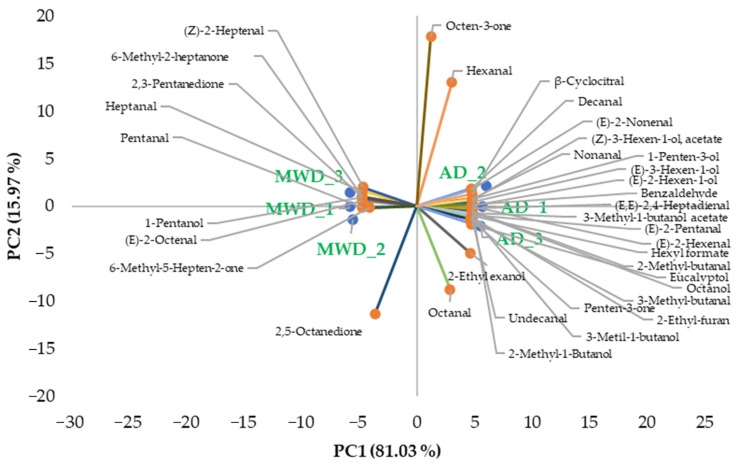
PCA loading plot of olive leaf herbal tea volatile aroma compounds. AD: herbal tea from air dried olive leaf. MWD: herbal tea from microwave dried olive leaf.

The PCA biplot (Figure 3) shows the distribution of samples and volatile compounds along the first two principal components, which together explain 97.01% of the total variance (PC1: 81.03%, PC2: 15.97%), indicating an excellent representation of the dataset in a two-dimensional space. A clear separation between the two treatments is observed mainly along PC1, suggesting that the treatment had a strong impact on the overall volatile profile.

MWD samples were distributed on the negative side of PC1, whereas AD samples are located on the positive side, highlighting distinct aroma fingerprints associated with each drying condition. Compounds contributing positively to PC1, and therefore associated with the AD samples, include several aldehydes, alcohols, and esters such as (*E*)-2-hexenal, (*E*,*E*)-2,4-heptadienal, decanal, undecanal, octanol, 2-methyl-1-butanol, 3-methyl-1-butanol, hexyl formate, and eucalyptol. These compounds are known to contribute to green, fatty, fruity notes. Conversely, the MWD samples are correlated with compounds such as pentanal, heptanal, (*Z*)-2-heptenal, 2,3-pentanedione, 6-methyl-2-heptanone, 1-pentanol, and (*E*)-2-octenal, indicating a different volatile profile, possibly related to alternative oxidation pathways or thermal degradation mechanisms. The PC2, although explaining a smaller proportion of variance, further differentiates specific samples and compounds. In particular, octen-3-one and hexanal load positively on PC2, while 2,5-octanedione and octanal show negative loadings, suggesting secondary variations in oxidative and ketonic compounds among samples.

Overall, the PCA biplot confirms that the applied treatments significantly influenced the volatile composition, leading to clearly distinguishable aromatic profiles. The strong separation and high explained variance underline that processing conditions play a key role in shaping the volatile fingerprint of the samples.

### 3.3. Sensory Analysis

#### 3.3.1. Qualitative Descriptive Analysis

**Figure 4:**
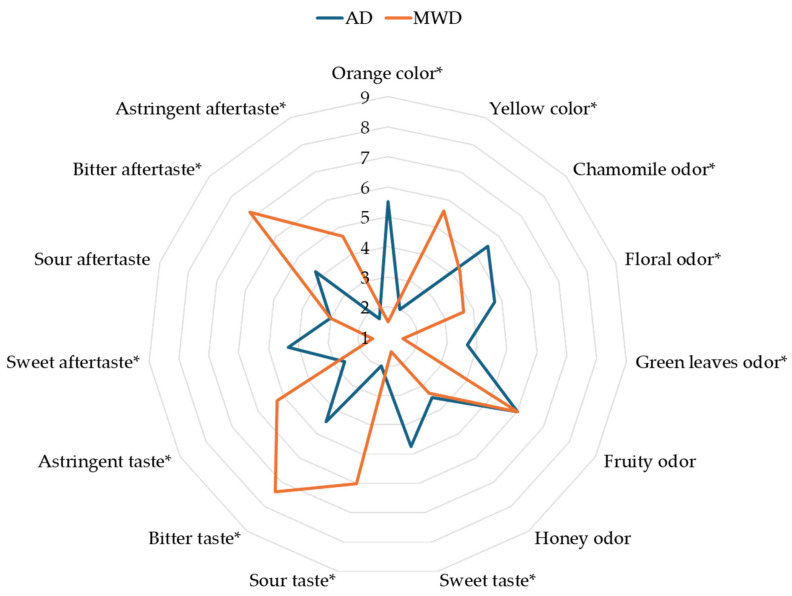
Graphical representation of the sensory profile of olive leaf herbal teas. AD: herbal tea from air dried olive leaf. MWD: herbal tea from microwave dried olive leaf. * Statistically significant differences at *p* < 0.05.

Sensory analysis revealed significant differences among olive leaf herbal teas across the evaluated sensory descriptors (Figure 4).

Furthermore, the color of the herbal teas was distinctive of the drying techniques used. AD herbal teas showed an orange color, whereas MWD samples showed a yellow color. This difference may be associated with the degradation of chlorophyll and carotenoid content, but also with enzymatic and non-enzymatic reactions during the drying process [47]. Studies have shown that long treatments and high temperatures can increase the alteration of these substances, changing their color [48,49].

The AD samples reported the highest intensity of most of the olfactory descriptors, particularly the odour of chamomile and green leaves. The olfactory profile can be associated with the differences found in the volatile fraction. In fact, the herbaceous, floral odors that characterized the AD samples are directly associated with the higher amounts of alcohols (in particular 2-methyl-1-butanol, 3-methyl-1-butanol, and (*Z*)-3-hexen-1-ol) and terpenes (β-cyclocitral and eucalyptol), which give green, fresh, and balsamic notes typical of olive leaves [50,51].

The taste descriptors of sour, bitter, and astringent characterized the MWD samples, while the AD samples had a sweet taste. A comparable trend was observed for the aftertaste descriptors, with the only exception being the sour aftertaste, where no differences were found between the samples. This difference could be associated with the higher content of total polyphenols, and consequently oleuropein, the most abundant phenolic compound in olive leaves and responsible for their bitter taste [52,53].

#### 3.3.2. Consumers’ Acceptability

**Figure 5:**
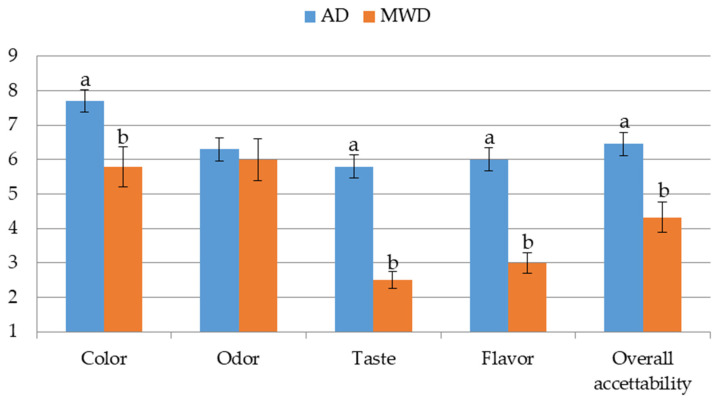
Consumers’ acceptability of olive leaf herbal teas. AD: herbal tea from air dried olive leaf. MWD: herbal teas from microwave dried olive leaf. Different letters for the same attribute indicate statistically significant differences at *p* < 0.05 by Duncan’s test.

Figure 5 shows the results of consumers’ acceptability of olive leaf herbal teas. Herbal teas produced from AD leaves were more preferred than those from MWD leaves. In particular, the largest difference between samples was observed for taste, with scores of approximately 5.8 for AD and 2.5 for MWD. This indicates that consumers preferred sweeter taste and less sour, bitter, and astringent characteristics, as highlighted by the descriptive analysis. The same trend was also observed for the flavour attribute.

The colour attribute highlights how consumers prefer herbal teas with orange tones; this suggests that colour changes associated with the degradation of chlorophyll and carotenoid content due to air drying did not influence overall acceptability. No significant differences were observed for the odour attribute between the samples, with values of ~6.0. This is probably because inexperienced tasters are influenced by the high fruity notes found in both samples analysed. All these data are reflected in a higher overall acceptability of the samples produced from AD leaves (6.5 vs. 4.2). The consumption habits expressed by participants during the selection phase made it possible to identify a diverse and equally representative sample of consumers. Furthermore, the standardized service methods and randomization of samples confirmed that the results obtained on acceptability were not influenced by external or confounding factors. The data from consumers’ acceptability studies are in contrast with other recent studies carried out on herbal teas produced from leaves using the same drying processes [25,26,27]; suggesting that AD, when applied to olive leaves, makes the olive herbal teas more appreciated. Furthermore, the results confirm that the effects of the drying technique can vary depending on the matrix to which it is applied [54]. Insights from the broader literature on microwave drying further support the interpretation of the present findings. In particular, studies on food matrices with different structural and moisture characteristics have shown that microwave energy strongly affects moisture diffusivity and drying kinetics, while also influencing temperature profiles within the product. Carvalho et al. [55], investigating microwave and microwave–vacuum drying of barley malt, demonstrated that variations in moisture content and dielectric properties govern the interaction with microwave energy, leading to different thermal and mass transfer behaviors compared to convective drying. Although barley malt differs substantially from olive leaves, these observations provide a useful comparative framework, suggesting that matrix-specific water distribution and heat dissipation mechanisms may partially explain the different effects of microwave drying observed across food materials.

## 4. Conclusions

The results of this study highlight the influence of drying technology on the quality attributes of olive leaf herbal teas, providing relevant indications for their industrial production. Although microwave drying proved effective in increasing total phenolic content, this advantage was not accompanied by a proportional enhancement of antioxidant capacity and resulted in less favourable sensory characteristics. Conversely, air drying preserved a more balanced volatile profile, maintaining alcohols, esters, and terpenes associated with herbal and floral notes, resulting in higher consumer acceptability. Air drying emerges as a technologically suitable and reliable processing method for olive leaf herbal tea production. Despite longer processing times compared to microwave drying, air drying offers greater control over aroma development and sensory quality, which are critical factors for consumer-oriented products such as herbal infusions. Moreover, air drying technology is widely available, scalable, and easily integrable into existing processing lines, facilitating the valorisation of olive leaves as a low-cost and sustainable raw material. Overall, these findings support the use of air dried olive leaves for the development of commercially viable herbal teas with high sensory appeal. The study contributes practical insights for the food and nutraceutical sectors and aligns with circular economy strategies by promoting the transformation of olive by-products into value-added functional beverages. This study represents a starting point for further research aimed at evaluating different olive cultivars and characterising their polyphenolic and volatile profiles in greater detail. While the present findings highlight the influence of drying processes on the volatile composition of olive leaves herbal teas, the lack of fully quantitative data represents a limitation in assessing absolute concentration changes and the aroma relevance of individual compounds. Future studies incorporating quantitative analyses of volatile and aroma-active compounds, including the determination of odor activity values (OAVs), may allow a more robust evaluation of processing-induced chemical modifications and facilitate the exploration of potential relationships between composition and sensory attributes. Such approaches would contribute to a deeper understanding of the impact of drying processes on sensory perception and support the assessment of potential applications of dried olive leaf extracts in the development of functional foods and nutraceuticals.

## Figures and Tables

**Table 1:** Volatile aroma compounds of the olive leaf herbal teas.

Compound	LRI ^1^	AD ^2^ %	MWD ^3^ %	Odour ^4^
*Alcohols*				
1-Penten-3-ol	1163	**0.14 ± 0.01 a ^5^**	**-b**	vegetable
2-Methyl-1-Butanol	1206	**2.10 ± 0.11 a**	**-b**	fatty, greasy
3-Methyl-1-butanol	1207	**2.75 ± 0.14 a**	**-b**	fruity, banana
1-Pentanol	1245	**-b**	**0.75 ± 0.05 a**	fermented
(*Z*)-3-Hexen-1-ol	1377	**5.10 ± 0.23 a**	**0.63 ± 0.02 b**	green, herbaceous
(*E*)-2-Hexen-1-ol	1382	**0.80 ± 0.04 a**	**0.23 ± 0.01 b**	fresh, fruity
2-Ethyl hexanol	1477	1.11 ± 0.07	1.76 ± 0.07	citrus
1-Octanol	1546	2.92 ± 0.12	2.50 ± 0.09	fatty-citrus
All		**14.92 ± 0.72 a**	**5.86 ± 0.24 b**	
*Aldehydes*				
2-Methyl-butanal	913	**4.40 ± 0.25 a**	**0.43 ± 0.02 b**	cocoa, musty
3-Methyl-butanal	916	**2.92 ± 0.18 a**	**0.72 ± 0.03 b**	aldehydic, fruity
Pentanal	977	**0.84 ± 0.03 b**	**4.98 ± 0.31 a**	fermented, fruity
Hexanal	1077	**2.83 ± 0.07 b**	**5.54 ± 0.42 a**	grassy, leafy, fatty
(*E*)-2-Pentanal	1165	**0.36 ± 0.01 a**	**-b**	fruity, apple
Heptanal	1177	**0.72 ± 0.03 b**	**3.21 ± 0.21 a**	aldehydic, fatty
(*E*)-2-Hexenal	1216	**2.84 ± 0.14 a**	**-b**	grassy, leafy
Octanal	1280	**6.74 ± 0.26 b**	**14.03 ± 0.48 a**	aldehydic, orange
(*Z*)-2-Heptenal	1321	**0.25 ± 0.01 b**	**0.83 ± 0.05 a**	-
Nonanal	1386	44.44 ± 1.87	43.73 ± 1.12	orange peel
(*E*)-2-Octenal	1428	**0.28 ± 0.01 b**	**1.54 ± 0.09 a**	fatty
(*E,E*)-2,4-Heptadienal	1465	**1.34 ± 0.09 a**	**-b**	fatty
Decanal	1493	**5.88 ± 0.26 b**	**7.03 ± 0.24 a**	aldehydic
Benzaldehyde	1530	**0.60 ± 0.02 a**	**-b**	fruity, almond
(*E*)-2-Nonenal	1535	**0.63 ± 0.04 b**	**1.02 ± 0.03 a**	fatty
Undecanal	1486	**0.21 ± 0.01 a**	**0.15 ± 0.01 b**	floral, citrus
All		**75.29 ± 3.28 b**	**83.21 ± 3.01 a**	
*Esters*				
3-Methyl-1-butanol acetate	1112	**0.39 ± 0.02 a**	**-b**	fruity
(*Z*)-3-Hexen-1-ol acetate	1307	**3.53 ± 0.11 a**	**1.21 ± 0.09 b**	fruity
Hexyl formate	1344	**0.85 ± 0.05 a**	**-b**	fruity
All		**4.78 ± 0.18 a**	**1.21 ± 0.09 b**	
*Furans*				
2-Ethyl-furan	950	0.54 ± 0.02	0.67 ± 0.04	malty
All		0.54 ± 0.02	0.67 ± 0.04	
*Ketones*				
Penten-3-one	1020	**0.36 ± 0.02 b**	**0.56 ± 0.03 a**	pungent, peppery
2,3-Pentanedione	1057	**-b**	**0.34 ± 0.01 a**	sweet, buttery
6-Methyl-2-heptanone	1231	**-b**	**0.27 ± 0.01 a**	fruity, citrus
1-Octen-3-one	1294	**0.42 ± 0.01 b**	**0.89 ± 0.05 a**	mushroom, hearty
2,5-Octanedione	1316	**0.63 ± 0.04 b**	**1.45 ± 0.09 a**	-
6-Methyl-5-hepten-2-one	1331	**2.20 ± 0.10 b**	**5.35 ± 0.13 a**	citrus, fruity
All		**3.61 ± 0.17 b**	**8.86 ± 0.32 a**	
*Terpenes*				
Eucalyptol	1195	**0.31 ± 0.01 a**	**0.13 ± 0.01 b**	eucalyptus
β-Cyclocitral	1625	**0.55 ± 0.03 a**	**-b**	hay-like, mild floral
All		**0.86 ± 0.04 a**	**0.13 ± 0.01 b**	

^1^ Linear retention indices calculated on a Vf-Wax-ms 60 m capillary column; ^2^ air dried olive leaf herbal tea; ^3^ microwave dried olive leaf herbal tea; ^4^ odor descriptors were identified by Flavornet Database https://www.flavornet.org/index.html (accessed on 15 December 2025); ^5^ different letters in the same row indicate significant differences (*p* < 0.05); in bold statistically significant differences (*p* < 0.05).

## Data Availability

The original contributions presented in this study are included in the article. Further inquiries can be directed to the corresponding author.

## Abbreviations

The following abbreviations are used in this manuscript:

AD	Air Drying
MWD	Microwave Drying
TPC	Total Phenolic Content
AC	Antioxidant Capacity
HS-SPME-GC-MS	Headspace Solid Phase Microextraction
BHT	Butylated Hydroxytoluene
BHA	Butylated Hydroxyanisole
DVB/CAR/PDMS	Divinylbenzene/Carboxen/Polysimethylsiloxane
GC-MS	Gas Chromatography-Mass Spectrometry
LRIs	Linear Retention Indices
QDA	Qualitative Descriptive Analysis
ANOVA	One-Way Analysis Of Variance
PCA	Principal Component Analysis
DPPH	2,2-Diphenyl-1-Picrylhydrazyl
MAE	Microwave-Assisted Extraction
